# Enhanced Co-Worker Social Support in Isolated Work Groups and Its Mitigating Role on the Work-Family Conflict-Depression Loss Spiral

**DOI:** 10.3390/ijerph13040382

**Published:** 2016-03-29

**Authors:** Wesley P. McTernan, Maureen F. Dollard, Michelle R. Tuckey, Robert J. Vandenberg

**Affiliations:** 1 Asia Pacific Centre for Work Health and Safety, University of South Australia, Magill 5072, Australia; maureen.dollard@unisa.edu.au (M.F.D.); michelle.tuckey@unisa.edu.au (M.R.T.); 2 Department of Management, Terry College of Business, University of Georgia, Athens, GA 30602, USA; rvandenb@uga.edu

**Keywords:** depression, work-family conflict, social support, mining

## Abstract

This paper examines a loss spiral model (*i.e.*, reciprocal relationships) between work-family conflict and depression, moderated by co-worker support. We expected that the moderation effect due to co-worker support would be evident among those working in isolation (*i.e.*, mining workers) due to a greater level of intragroup attraction and saliency attributable to the proximity effects. We used a two wave panel study and data from a random population sample of Australian employees (*n* = 2793, [*n* = 112 mining, *n* = 2681 non-mining]). Using structural equation modelling we tested the reciprocal three way interaction effects. In line with our theory, co-worker support buffered the reciprocal relationship between WFC and depression, showing a protective effect in both pathways. These moderation effects were found in the mining industry only suggesting a proximity component moderates the social support buffer hypothesis (*i.e.*, a three way interaction effect). The present paper integrates previous theoretical perspectives of stress and support, and provides insight into the changing dynamics of workplace relationships.

## 1. Introduction

### 1.1. Social Support and Work-Family Conflict among Remote Workers

Social support is typically sought from and provided by primary groups: friends, family members and partners. Different types of primary group support, in particular spousal support, have been shown to be predictive of outcomes of life satisfaction as well as positive and negative mood [1]. Social support has also been theorized as a key moderator of the experience of stress in both the psychology [2,3,4] and sociology literature [5,6,7,8]. It therefore plays an important role in dealing with daily stress, such as the stress from work, through the informal support structures typically offered by partners, friends and family.

However, a recent trend in the work arrangements adopted by primary resources industries may restrict the availability of these support networks. Fly-in/Fly-out (FIFO) rosters, where a worker is flown to a remote worksite to stay and work for a period of days and return home for rest days, has become a prevalent strategy for remote employment in the mining industry as well as offshore oil and gas industries. FIFO rosters have become especially popular among Australian mining employers, due to the great distances between coastal urban populations and inland mining sites. Employees, particularly those with partners and children, prefer FIFO contracts over relocating to mining communities that are closer to mining sites but these lack the infrastructure of Australia’s major urban populations.

This relocation however restricts both the support a worker can receive from primary groups, as well as the support they too can offer. This would suggest that Australian mining workers, in particular those on FIFO contracts, will experience greater conflict between their work and family domain. Recent research in the Australian Workplace Barometer project supports this, with mining workers reporting the highest rates of work-family conflict across all Australian industries [9]. Work-family conflict has been theorized as a construct that elicits a stress response (a stressor) [10], which can lead to impairments in psychological health such as depression, and also a product of stress [11]. In this regard, the relationship between work-family conflict and psychological health may be bidirectional. Problematically, remote mining workers do not have access to their primary support networks that are important for buffering this stress process. However, in the absence of their typical support networks, we drawn on the *proximity principle* to suggest that remote mining workers will seek social support from those around them on site, *i.e.*, their co-workers. In the present paper, we therefore propose that a bi-directional relationship between work-family conflict and depression will be mitigated by co-worker support among mining workers.

### 1.2. Work-Family Conflict and Mental Health

Work-family conflict is associated with a myriad of psychological health impairments. Work-family conflict, also referred to as *work-home conflict* is an antecedent of psychological health including outcomes such as depression [10,12], wellbeing [13], burnout [14], and exhaustion [15]. We can explain this phenomenon through the stressor-strain process: the experience of work-family conflict elicits stress in an individual that if not addressed leads to psychological strain related outcomes, *i.e.*, a decline in psychological health and wellbeing. This process is an underlying assumption in contemporary work stress theories in psychology such as Karasek’s Job Demand Control Model [3] and more recently Demerouti and Bakker’s Job Demand Resources model [4], as well as popular sociological theory on stress such as Pearlin’s stress-process model [16].

Conversely, there is also evidence to suggest that conflict between work and family domains can be an *outcome* of stress. Research by Kelloway, Gottlieb, and Barham [11] found that an increase in work-family conflict was significantly predicted by the perceived psychological stress of employees. One explanation Kelloway, *et al.* propose is that our personal resources (*i.e.*, emotional and physical energy) are depleted in response to stressors, which in turn leaves us with inadequate resources to cope with the conflict between work and family life. Moreover a meta-analysis by Allen, Herst, Bruck and Sutton [17] found work-family conflict predicted stress and stress-related outcomes. This incongruence could be evidence of bidirectional relationship between stress-related outcomes (such as generalised stress, or specific impairments like depression) and work-family conflict, where initial conflict acts as a stressor eliciting strain, which in turn depletes resources that are also needed to address the conflict between the work and family domain.

Adding support to this proposition, Demouriti, Bakker and Bulters [15] explicitly explored work-family conflict as a stressor and strain outcome concurrently. They found that work-family conflict interacted with emotional exhaustion in a negative spiral similar to Hobfoll’s [18] loss spiral theory. According to Hobfoll, a person’s resources are taxed when addressing stressors. Once a person’s resources are low, they are then less equipped to deal with subsequent stressors, and consequently less equipped to address the initial stressor at its next occurrence. In this sense, resources are depleted in “loss spirals” and conversely can be gained in “gain spirals”. Similarly, Demouriti *et al.* [15] argued that a person who is exhausted would have insufficient energy to address work-family conflict, which in turn acts as an additional source of strain, creating more exhaustion in a loss spiral. These findings echo previous work by Leiter and Durop [19] who similarly found a reciprocal relationship over time between work-family conflict and exhaustion.

**Figure 1:**
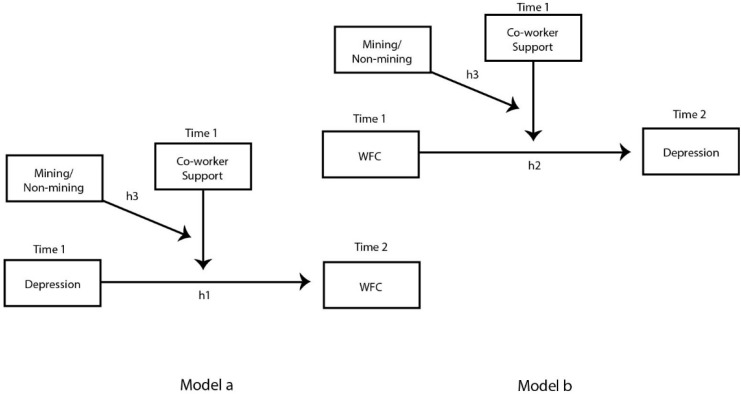
Buffering hypothesis of a Work-Family Conflict/Depression loss spiral. WFC = Work-Family Conflict; h1-3 indicate hypotheses tested.

Considering the loss spiral found by Demouriti *et al.* [15] a similar spiral is plausible with other strain-related outcomes that relate to heightened levels of work-family conflict, such as depression [10,12]. We propose that a person experiencing depression will have insufficient energy to address conflict between work and home, which will in turn act as a source of stress further exacerbating depression. We therefore we hypothesize:

Hypothesis 1:  Depression increases the level of work-family conflict (model a, Figure 1).

Hypothesis 2:  Work-family increases the level of depression (model b, Figure 1).

### 1.3. The Social Support Buffer Hypothesis

In the absence of their usual support network of friends, family and partners, remote mining workers may be at a greater risk of strain-related health outcomes that arise from the experience of stressors. Within the literature of psychology, social support is regarded as a critical resource in protecting mental health, and has been argued to act as a *buffer* of psychological stress in the prominent work of research contemporaries such as Cohen and Wills [2], Karasek and Theorell [3] and Bakker and Demerouti [4]. In one of the earliest papers to discuss the social buffer effect, Cohen and Wills proposed social support may function as a coping mechanism against an elicited stress response by preventing the initial stress appraisal, in light of the available peer support. They also proposed that social support may aid in preventing the stress response by functioning as a resource that provides alternate means to address the stressor. Adding additional support Frese [20] observed the support buffering hypothesis—that is, the buffering effect of social support against stress—amongst a range of different stressors (e.g., physical and psychological) and outcomes (e.g., depression and anxiety) with different forms of support (e.g., co-worker and spousal).

Similar perspectives of stress and support are proposed in the sociology literature, but provide additional qualitative perspectives on the mechanism through which the stress response occurs. Pearlin’s [16] *stress process model* proposes that the stress response that arises from the experience of a stressor can lead to negative self-appraisal, affecting self-esteem and our sense on control of life events. Pearlin proposes that through this mechanism, poor mental health, in particular depression, can arise. Further, social support has been argued to play a vital role in protecting mental health in buffering the stress process in Pearlin’s model [5,6,7,8]. Social support has been argued to buffer the stress response in that support networks provide resources to cope with the occurrence of stress. Together these perspectives not only reflect theoretical convergence across disciplines, but a complimentary account of the stress and social support experience. We hereon refer to this process as the *social support buffer hypothesis*.

However, in the absence of their usual support network, it is plausible that remote workers will seek social support from their co-workers. We offer two theoretical perspectives to support this proposition. First, the proximity principle suggests remote mining workers will be at a greater disposition to seek social support from co-workers in the absence of friends and family. In the field of social psychology the proximity principle refers to the increased likelihood of people forming social bonds and establishing group cohesion when they are in frequent physical contact. Since the 1960s research has investigated the phenomenon in terms of geographical proximity, largely in studies of friendship formation amongst university students living in campus boarding facilities [21,22,23], but the theory has also been investigated in terms of interpersonal similarity as a form of proximity [24]. Proximity in university dorm-rooms was additionally associated with increased disclosure [23], which may facilitate supportive discussion. This finding would suggest that workers in isolated environments such as remote miners, who have more social interactions with co-workers than typical, and less with friends and family at home, are increasingly likely to receive and rely upon social support from their co-workers.

Second, more alleviative co-worker social support in isolated workplaces could also be explained by processes of self-categorisation. According to self-categorisation theory [25], the personalities of members of a collective are influenced by the behaviours and social norms of that group. These prototypical behaviours of the group form a “social identity” for the individual. Prototype behaviours typically are those that emphasise characteristics of the group as well as behaviours that differentiate group members from other groups. For example, a person working in the agricultural industry may wear clothing that reflect his profession outside of work, that both associate him with the agriculture industry as well as distinguish him from a person in an office job role. Reflecting prototype behaviour enhances group saliency, intragroup social attraction and overall group cohesion [26]. The greater frequency that remote mining workers interact should therefore result in a greater number of prototype behaviours and a stronger social identity, and consequently, greater intragroup cohesion and stronger social relationships. Self-categorisation theory, therefore, provides an additional explanation for strengthened social relationships amongst remote mining workers, which should in turn buffer strain. Both of these theoretical perspectives support the notion of a proximity component to social support, which we propose will result in a social support buffering effect among a mining cohort. We therefore propose:

Hypothesis 3:  Co-worker support amongst mining workers reduces or buffers the bidirectional relationship between work-family conflict and depression.

The following model is proposed to test our hypotheses:

### 1.4. Aims and Significance

This paper aims to construct a model that unifies two predominant perspectives within the literature, the social support buffer hypothesis and loss spiral theory. Further, we elaborate on the social support buffer hypothesis by exploring the environmental factor of workgroup proximity, which should mitigate the efficacy of social support according to the proximity principle, and self-categorizing processes. The inclusion of mining as a group factor has additional important practical implications for industry. The mining industry plays a vital component of world economies, in particular the mineral-driven economies of Australia, South Africa, Canada, Russia, and the United States.

Examining depression has potential practical implications for industry practices as well. Depression is associated with memory [27] and decision-making [28] impairments. With the high rates of traumatic workplace injuries in the mining industry [29], research that can inform strategies to alleviate stress-related illnesses with cognitive correlates stands to improve site safety, as well as staff wellbeing. Additionally, depression is associated with several productivity factors such as presenteeism and sickness absenteeism, that have financial implications for the employer [30,31]. Considering these safety and performance correlates, this study may be beneficial in improving our understanding of the pathways of work-related stress, to inform organisational policies and practices, and in turn improve the health of employees, companies, and their related economies.

## 2. Methods

### 2.1. Procedure and Participants

This study uses survey data collected as a part of nation-wide surveillance project of work stress factors, the Australian Workplace Barometer project. We used a repeated measures design across two time points of data collection with a 12-month time lag, between 2009 and 2010 from New South Wales and Western Australia, and between 2010 and 2011 for South Australia. Ethics was obtained from the University of South Australia’s Human Research Ethics Committee. The sample consisted of 2793 (48.3% male, 51.7% female) working Australians between the ages of 18 and 85 who completed the survey at both time points. Within our sample 112 (83.9% male, 16.1% female) were identified as working in the mining industry which was used to create the dichotomous variable regarding whether they were mining workers or not (see measurement description below).

Telephone interviews were conducted with participants. To recruit participants, phone numbers were randomly selected from the Australian white pages telephone directory for each state. After contact was made, the interviewer asked to speak to an employed member of the household over 18 years of age and who had the most recent birthday. It was this individual who was then invited to participate.

### 2.2. Measures

Depression was measured using the nine-item Patient Health Questionnaire (PHQ-9). The nine items reflect the criteria for Major Depressive Disorder used in the DSM-IV [32] e.g., “During the last month, how often were you bothered by feeling down, depressed or hopeless?” Responses range from 0 (not at all) to 3 (nearly every day). Work-Family Conflict was measured using Netemeyer, Boles and McMurrian’s [33] five-item measure. Items reflected the negative spill over from work into the family domain, e.g., “My job produces strain that makes it difficult to fulfil family duties”. Responses were made on a seven point Likert type scale ranging from 1 (strongly disagree) to 7 (strongly agree). The fifth item was removed due to poor factor loading.

Co-worker support was measured using the co-worker support subscale from the Job Content Questionnaire 2 [34]. The measure of co-worker support consisted of 3 items, e.g., “I am treated with respect by my co-workers”. Participants responded on a 4 point Likert type response scale ranging from 1 (strongly disagree) to 4 (strongly agree).

Participants reported the industry they worked within, giving responses classified under the Australian and New Zealand Standard Industrial Classification (ANZSIC). These responses were recoded into a dichotomous measure indicating whether or not the participant worked within the mining industry (2) or not (1).

### 2.3. Data Analyses

Using MPlus version 6.11 [35], a Structural Equation Model (SEM) was conducted using a full two-wave panel design. Missing responses were replaced by the series mean of participant scores. To test Hypothesis 1 and 2: four models were constructed: a stability model; model a (WFC, Co-worker Support, Mining → Depression); model b (Depression, Co-worker Support, Mining → WFC) and a reciprocal model (a and b).

Once the model with the most appropriate fit was established, the interaction terms were added to the model to test hypothesis 3 (the moderating effect of co-worker support amongst mining workers). When modelling interaction effects in SEM, standard fit indices are inapplicable. Therefore alternative measures of fit, The Akaike Information Criterion (AIC) and the Sample-Size Adjusted Bayesian Information Criterion (SABIC) were examined to measure the fit of the interactions added to the reciprocal model. See Little, Bovaird and Widamen [36] for a discussion on the use of fit indices when modelling interaction terms. We chose to use SEM for our analysis because it allowed us to test both causal models simultaneously, as well as allowing us to model the additional complex interaction terms.

## 3. Results

### 3.1. Descriptives

Before conducting the analysis, means, standard deviations, Cronbach’s alphas (Table 1) and correlations (Table 2) were computed. All variables showed satisfactory internal consistency (between 0.81 and 0.90). Both WFC and depression had a test-retest reliability of >0.50, and co-worker support of >0.30. These reliability coefficients were all statistically significant and of typical stability for this type of research.

### 3.2. Analysis

Table 3 shows the comparative models tested: model a, model b, as well as testing both models simultaneously. It should be noted that in all four models, the chi square values were significant. This is nearly always the case for large models with large samples (400 or more cases) in which case researchers should rely on fit indices for model interpretation [37]. The fit indices (RMSEA, TLI and CFI) were sound for each model tested, however the reciprocal model yielded a lower chi square indicating it was a better overall fit, supporting Hypotheses 1 and 2. Interaction terms were then added to the reciprocal model.

**Figure 2:**
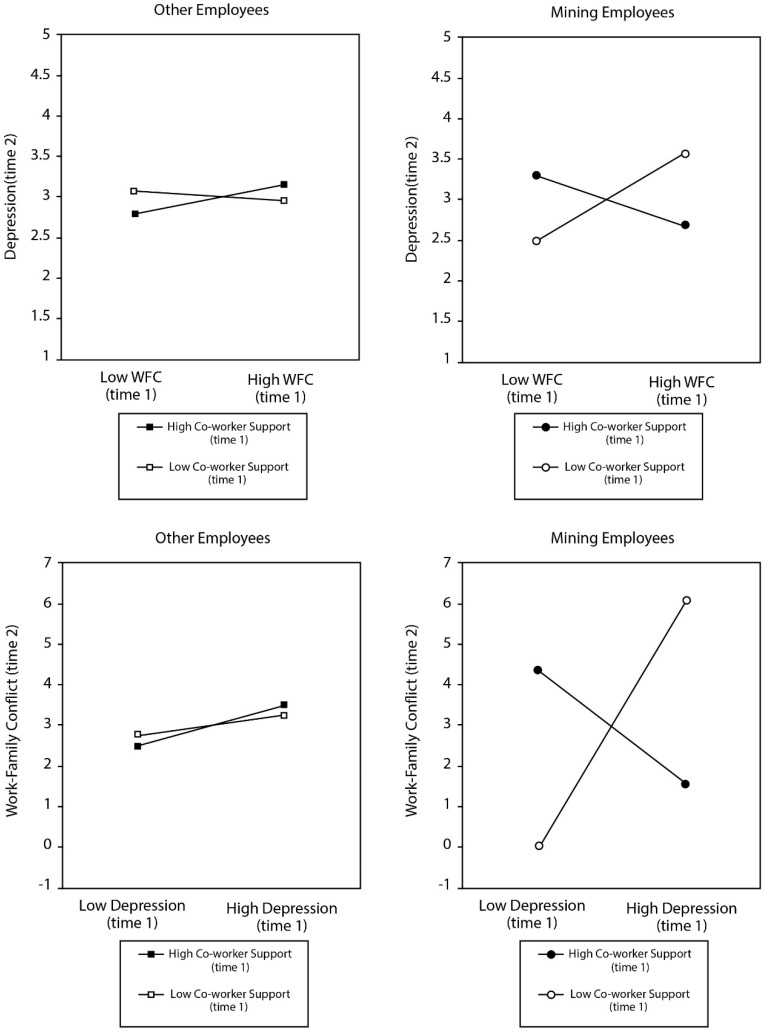
Interaction terms for the moderating effect of co-worker support on the work-family conflict depressive loss spiral in mining and a general work sample in Australia. *WFC = Work-Family Conflict*.

The inclusion of the interaction terms (Hypotheses 3) yielded a lower AIC (168,315.94) than the reciprocal model (168,323.65) and a SABIC (168,685.13) not notably greater than the reciprocal model (168,684.44), suggesting the inclusion of the interaction terms creates an equal if not better overall model fit. Interaction terms are displayed in Figure 2.

**Figure 3:**
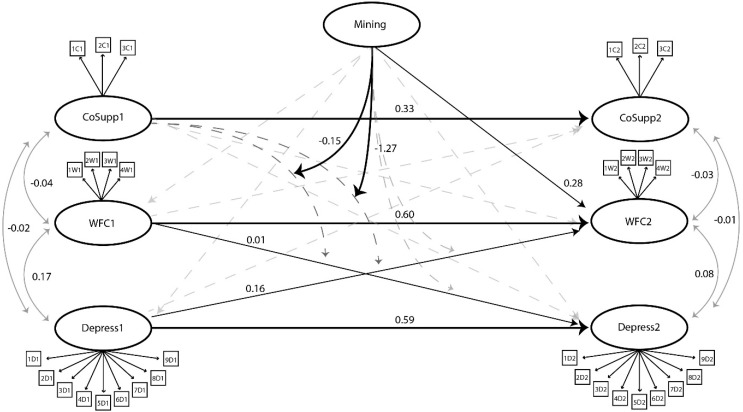
A two wave panel design structural equasion model with two and three-way interaction terms. CoSupp = Co-worker Support. WFC = Work-family Conflict. Depress = Depression. Dashed lines represent non-significant main effects.

Figure 3 shows the regression coefficients (*B*) of the modelled regression paths and correlations of our hypothesized model. Non-significant pathways are represented by dashed lines. Hypotheses 1 and 2 were confirmed, showing that Depression at time 1 significantly predicted an increase in WFC at time 2 (*B* = 0.16), and to a lesser extent WFC at time 1 significantly predicted and increase in Depression at time 2 (*B* = 0.01). The two way interaction was non-significant, showing co-worker support did not moderate the regression path from depression to WFC over time or WFC to depression over time. However, the three way interactions were significant confirming Hypothesis 3, showing mining strongly moderated the interaction of co-worker support on the regression path between depression and WFC (*B* = −1.27) over time and WFC to depression (*B* = −0.15) over time.

## 4. Discussion

The purpose of the present paper was to expand the current theoretical knowledge of stress processes by proposing a unified model of stress by modelling two prominent theoretical concepts simultaneously: loss spiral theory, and the social support buffer hypothesis which is common among psychological and sociological stress theory. Further, we extend the theoretical understanding of social support by exploring proximity effects via the additional interaction of remote work (mining) in this integrated model. Our study hypotheses were supported, finding a reciprocal relationship between depression and WFC, which was buffered by social support only among mining workers. Theoretical and practical contributions are discussed.

First, in finding a reciprocal relationship between work-family conflict (WFC) and depression this paper adds to the emerging body of research on Hobfoll’s [18] growth and loss spiral theory, which has implications for current stress process perspectives. In particular, this finding helps account for a gap in current stress process perspectives, as loss spiral theory could explain the incongruency in previous research regarding WFC’s role as either a stressor or an outcome of stress. Loss spiral theory suggests WFC can act as both a cause and outcome of stress. Personal resources are depleted in addressing the stress caused by WFC, which leads to insufficient resources to respond to another stressor (*i.e.*, depression). Resources are further taxed to respond to that stressor, which results in less resources respond to WFC in the future, creating a loss spiral. Similarly depression functions as a stressor as symptoms of depression (such as lethargy and anhedonia) make it difficult to engage in both work and social roles. Personal resources are depleted in addressing this elicited stress, leading to insufficient resources to address the experience of WFC. Hobfoll’s theory suggests that unidirectional assumptions on the experience of stress may not always correct, and therefore the current prevailing perspectives on the stress process (*i.e.*, the Karasek’s JDC theory [3], Bakker’s JDR theory [4] and Pearlin’s stress process theory [16]) could be expanded to account for reciprocity between stressors and stress outcomes.

There are also considerable implications of the two way interactions being non-significant and the three-way interactions being significant (Hypothesis 3). Our findings suggest that co-worker support significantly acted as a buffer of work-family conflict within the mining working population, but not significantly in the general working population. This supports our hypothesis that there is a proximity component to the buffer hypothesis, that proximity mitigates the efficacy of social support. Extrapolating on this hypothesis, not only does proximity enhance the effect of social support, but the antithesis seems to also be evident, in that distance appears to be deleterious on social relationships. This is evident in a significant pathway between mining at Time 1 to work-family conflict at Time 2 (*B* = 0.28).

These findings may serve to explain the incongruent findings for and against the social support buffer hypothesis present in the literature. Although several studies have found small to moderate support for the buffer hypothesis among work cohorts [20,38,39], several studies have found no effect [40,41]. As outlined in the introduction, we believe that the increased proximity that mining colleagues experience, who are often on FIFO or similar contracts, enhances the social cohesion amongst co-workers and the strength of their social bonds, therefore enhancing the protective strength of social support as a buffer. It is plausible that cohorts used in previous research differed in both intra-group proximity, which will affect the efficacy of co-worker social support in alleviating stress.

There is the additional possibility that part of the moderation could be attributed to gender effects. Ystgaard, Tambs and Dalgard [42] found the social support buffer present amongst male adolescents but not female. Ystgaard *et al.* speculated that this gender difference may be attributable to traditional gender roles: that male friends will disperse more problem solving advice and that female friends will provide a more passive support role, and that the former may be more effective. As the 83.9% of the mining worker respondents were male, a larger male population could explain the relationship. To rule out this possibility, we conducted a post-test analysis replicating model using gender instead of mining as the third interaction term. The relationship was shown to be non-significant at both model a. and model b., adding further support to our hypotheses.

A practical implication of these findings is that a means for minimising the WFC-depression loss spiral is identified for mining workers, and may also be generalizable to other isolated working populations. Organisational policies and practices that foster supportive and cohesive co-worker relationships may be able to help minimise depression and WFC. Considering the associated productivity cost deficits associated with depression [31], there may be considerable financial benefits by co-worker support targeted organisational practices. Further, mining workers represent a critically under-examined population in terms psychological health despite their contribution to substantial economies. This paper therefore contributes to a gap in empirical research which has been noted in previous literature [43].

While the two-wave design enabled reciprocal relationships to be examined over time, a third time wave would have more definitively elucidated the changes in these relationships over time. It is also possible that the period of at home and away time experienced by remote workers could affect the degree of conflict between the work and family domain, as the length of FIFO rosters can vary considerably. Additionally, factors such as telecommunication facilities (e.g., phone reception and internet services) and the remoteness of the workplace could also affect the experience of work family conflict. By examining these additional work context variables, future research could identify which workplace factors are more pertinent to the experience of work-family conflict amongst remote workers.

Another limitation was that the number of mining workers on FIFO contracts, was not identifiable. It is possible that those on FIFO contracts may experience a greater reliance on co-worker support than those living in neighbouring communities. However, it is likely that those that were not on FIFO contracts still experience remoteness as they live away from major urban populations, and still work similar hours. Further, the supportive culture that arises due to FIFO rosters is likely to spill-over to non-FIFO employees who they work with. Future research should explore the effect of roster type and length to see if this affects employee’s experience of co-worker support. A larger sample of mining workers would also be beneficial to obtain ample power to explore these roster-related effects on the experience of co-worker support.

## 5. Conclusions

In conclusion, the present paper offers a unification of stress process perspectives, and challenges unidirectional assumptions between stressors and strain-outcomes. Additionally the unique role of proximity may explain previous discrepancies in the literature, and therefore warrants consideration in future research design. As the way that we work changes, the effects that these changes have on our social relationship both at home and at work, warrants increasing attention.

## Figures and Tables

**Table 1:** Means, standard deviations (SD) and Cronbach’s alpha (α) for Australian employees.

Measure	Mean	SD	α
1. Co-worker support 1	9.79	1.29	0.87
2. Co-worker support 2	9.70	1.20	0.88
3. WFC 1	14.51	6.94	0.90
4. WFC 2	14.41	6.64	0.90
5. Depression 1	3.46	3.72	0.81
6. Depression 2	3.21	3.50	0.82

Mean-centering was conducted in the analysis before generating interaction terms. Numbers after variable name indicate time wave. *N* = 2793.

**Table 2:** Correlation matrix of study variables for Australian employees.

Measure	1	2	3	4	5
1. Co-worker support 1	−				
2. Co-worker support 2	0.31	−			
3. WFC 1	−0.06	*NS*	−		
4. WFC 2	−0.05	−0.05	0.60	−	
5. Depression 1	−0.10	−0.06	0.28	0.22	−
6. Depression 2	−0.08	−0.08	0.21	0.28	0.53

NS indicates non-significant correlations; otherwise all other statistics are significant to 0.05 or less. *N* = 2793.

**Table 3:** Main effects and fit indices for study models over time for Australian employees.

Model	*Main Effects*	*B*	*p*	*x*^2^	*df*	*p*	RMSEA	TLI	CFI
1. Stability model				2595.29	469	<0.001	0.040	0.944	0.950
2. Model a	WFC → Depression	0.01	<0.001						
	Co-worker Support → Depression	−0.02	ns						
	Mining → Depression	0.03	ns						
				2584.35	465	<0.001	0.040	0.943	0.950
3. Model b	Depression → WFC	0.18	0.02						
	Co-worker Support → WFC	−0.01	ns						
	Mining → WFC	0.26	0.03						
				2584.14	465	<0.001	0.040	0.943	0.950
4. Reciprocal model	WFC→ Depression	0.01	<0.001						
	Co-worker Support → Depression	−0.02	ns						
	Mining → Depression	0.05	ns						
	Depression → WFC	0.17	0.02						
	CoSupport → WFC	−0.02	ns						
	Mining → WFC	0.29	0.02						
				2573.59	462	<0.001	0.040	0.943	0.950

B = unstandardized beta coefficients. x^2^ = chi square, df = degrees of freedom, *p* = probability, RMSEA = Root Mean Square Error of Approximation, TLI = Tucker Lewis Index, CFI = Comparative Fit Index. *N* = 2793.
